# The role of the family physician in the fight against Coronavirus disease 2019 in Nigeria

**DOI:** 10.4102/phcfm.v12i1.2492

**Published:** 2020-06-18

**Authors:** Tijani I.A. Oseni, Ramatu O. Agbede, Bolatito B. Fatusin, Michael A. Odewale

**Affiliations:** 1 Department of Family Medicine, Faculty of Clinical Sciences, Ambrose Alli University, Ekpoma, Edo State, Nigeria; 2 Department of Family Medicine, Irrua Specialist Teaching Hospital, Irrua, Nigeria; 3 Department of Family Medicine, Ahmadu Bello University Teaching Hospital, Zaria, Nigeria; 4 Department of Family Medicine, Federal Medical Centre, Gusau, Zamfara State, Nigeria; 5 Department of Family Medicine, Faith Mediplex, Benin City, Edo State, Nigeria

**Keywords:** COVID-19, pandemic, Nigeria, family physicians, frontline

## Abstract

The Coronavirus disease 2019 (COVID-19) pandemic has been ravaging Nigeria and the world with increasing morbidity and mortality. Despite efforts by the Nigerian government implemented through the Nigerian Centre for Disease Control (NCDC) to reduce the scourge of the disease through public enlightenment and regular updates, the number of new cases and mortalities from COVID-19 are still increasing. Family physicians (FPs) who are the first contact of care for most patients accessing private and public health facilities in Nigeria have been working tirelessly to reduce the scourge of the pandemic in Nigeria. They continuously update themselves through regular webinars and online resources and guidelines provided by the Society of Family Physicians of Nigeria (SOFPON). Measures adopted by FPs across the country in the fight against the scourge include triaging patients as they present to the family medicine clinics; health education and enlightenment of the populace; and ensuring social distancing, regular handwashing and compulsory use of face mask by both physicians and patients during clinical consultations. Other measures include incorporating family-focused behavioural interventions in their practice, home-based care to reduce the number of persons visiting the hospital, telemedicine and Hospice and palliative care services to the elderly and terminally ill. In conclusion, FPs in Nigeria are helping to reduce the scourge of COVID-19 through patient education and innovative healthcare delivery that does not put patients at increased risk of the disease whilst promptly recognising potential COVID-19 patients and referring them for early diagnosis and treatment.

## Introduction

The Coronavirus disease 2019 (COVID-19) pandemic poses a major threat to Nigeria and Africa, with the dearth of health facilities across the continent.^1^ Mortalities from the disease have been on the rise worldwide, particularly in patients with comorbidities.^1^ Nigeria reported its first case on 28 February 2020^2^ and recorded the first COVID-19 mortality on 21 March 2020.^3^

The presence of the COVID-19 pandemic in Nigeria has caused unprecedented panic and confusion. The government through the Nigerian Centre for Disease Control (NCDC) made significant attempts to address this through daily updates on the morbidity and mortality patterns of the disease across states as well as measures to contain the disease, such as proper use of face mask, regular handwashing, social distancing and restriction of movement in the country. They also send out frequent SMS messages to the populace. However, the compliance of the general public with these measures is still minimal as some of them are still in denial.

## Family physicians and Coronavirus disease 2019

Most patients including those with respiratory symptoms usually present first to a primary healthcare, or out-patient clinic of tertiary hospitals.^4,5,6^ In Nigeria, family physicians (FPs) and family medicine trainees who are in charge of Family Medicine Clinics that include General Outpatient Department (GOPD) and Staff/National Health Insurance Scheme (NHIS) Clinics in tertiary health facilities, are often the first contact for patients presenting with features of COVID-19. Therefore, it is important that the role of FPs in the fight against COVID-19 should be recognised. This is particularly important considering the need for strong community-based efforts aimed at detecting and controlling local transmission of COVID-19.^4^

The management of COVID-19 requires constant surveillance, prompt diagnosis and early treatment.^3,6^ Thus, FPs being the first point of call in most hospitals have developed a high index of suspicion that help them promptly recognise patients with features suggestive of COVID-19 or patients exposed to COVID-19 patients or their contacts.^6,7^ They are also engaged in following regular updates through online webinars and courses as well as using resources and information from NCDC and the World Health Organization (WHO).

## Mitigating the effect of Coronavirus disease 2019 in family practice

The national body of FPs in the country, the Society of Family Physicians of Nigeria (SOFPON), had put measures in place to contain the spread of the disease whilst protecting themselves. These were written into an electronic guideline accessible to all FPs across the country in both public and private practice.^6^ Such measures are discussed below.

### Triaging

Triaging patients as they present to the family medicine clinics in both public and private practice by nurses helps separate patients with symptoms suggestive of infection with COVID-19 and those with travel history to or contact with person from an endemic area to be seen in separate consulting rooms from others. Thus, those with high likelihood of having contracted the disease are not seen in the regular clinics but seen at special clinics that are equipped with the provision of Personal Protective Equipment (PPE).

### Patient education and counselling

Education and enlightenment of the populace through health talks to patients presenting to family medicine clinics, distribution of handbills to the public and through print and electronic media are also an important function of the FP during this lockdown period. This has helped dispel rumours and provide accurate information on what should be done to prevent the disease and what to do if there is a suspected case. This has resulted in increasing number of persons coming for testing and greater compliance with preventive measures.^8^

### Conducive clinics and waiting areas

Family physicians in most facilities in Nigeria ensured that waiting areas were not overcrowded (patients sitting at least 1 m apart from each other in well-ventilated areas), running water with soap and hand sanitisers were provided at strategic locations within and outside clinics, appropriate clothing was worn (PPE where available, masks, goggles, hand gloves, etc.) and both the physician and patients wore face masks during clinical consultations.

### Family-focused behavioural intervention

Nigeria like most countries in Africa and the world is currently in lockdown.^3^ This poses a great challenge for families, particularly those who rely on daily earnings. Families being together at home for most times also have its attendant effect on family functionality. There have been increased cases of overcrowding, domestic violence. This has the potential of affecting both medical aspects and psychosocial well-being of families. Family-focused behavioural interventions such as family conferences have been shown to be effective in preventing complex health problems with social and behavioural components.^7^ Family physicians in Nigeria have also incorporated this in most of their practice.

### Home-based care

Family physicians in Nigeria are currently following home-based care to a greater extent than before. Home-based care is mainly intended for those who can afford it and more patients are opting for home-based care to avoid coming to the hospital. This has reduced the number of persons visiting the hospital, thus reducing the risk of contracting or spreading the disease. However, appropriate protective measures must be taken during such visits.

### Telemedicine

Telephonic and online conversations will also minimise the need of patients coming to hospital, thus minimising the spread of infection. Family physicians in most centres in the country have provided dedicated numbers or other means through which they could be reached by patients. They then identify those who need to come to the hospital. Telephonic appointments have also eliminated overcrowded waiting areas as patients are told when they are required to come. Over 60% of patients would be able to carry out telephonic consultations with their FPs.^6^

### Hospice and palliative care

The family physician provides holistic and comprehensive care.^6^ This has been strengthened during the COVID-19 pandemic by providing hospice and palliative care services to the elderly and terminally ill. This can be integrated into the pandemic plan.^1^ This can be further improved upon by being made flexible by providing protocols for disease management, training of caregivers and providing appropriate technology including telemedicine to minimise the contact and promote social distancing.^1^

### Challenges

Some of the interventions stated above, such as home visit and Hospice care, increase the cost of healthcare to patients and are mostly available to those who can afford it. This is particularly so as NHIS coverage in Nigeria is still low, with about 5% of the population enrolled.^9^ Hence, most patients still pay out of pocket both in private and public health facilities in the country.

## Conclusion

With the rising cases of COVID-19 in Nigeria, FPs as frontline doctors are helping to reduce the scourge through patient education and innovative healthcare delivery that does not put patients at increased risk of the disease whilst promptly recognising potential COVID-19 patients and referring them for early diagnosis and treatment.
